# Comprehensive Plasma Metabolome for Identification of Novel Biomarkers of Acute Myocardial Infarction

**DOI:** 10.1002/mco2.70303

**Published:** 2025-07-27

**Authors:** Jun Liu, Yi Yu, Qiqi Lu, Shan‐Qiang Zhang, Ji‐Cheng Li

**Affiliations:** ^1^ Major Disease Biomarker Research Laboratory School of Basic Medical Science Henan University Kaifeng China; ^2^ Institute of Cell Biology Zhejiang University Hangzhou China; ^3^ Department of Anatomy Shantou University School of Medicine Shantou China

**Keywords:** AMI, diagnosis, metabolomics, prognosis, UPLC‐MS/MS

## Abstract

Metabolic disorders play a crucial role in the occurrence of acute myocardial infarction (AMI). The objective of this research was to elucidate the characteristic metabolic profile of AMI and provide potential biomarkers for AMI. This study employed a targeted metabolomics approach utilizing the Ultra Performance Liquid Chromatography Tandem Mass Spectrometry (UPLC‐MS/MS) system to identify both hydrophilic and hydrophobic metabolites present in plasma samples. Among 1498 detected metabolites, 78 were the most significantly expressed in the AMI group. Functional synergy analysis showed prominent enrichment in the pathways of steroid hormone biosynthesis, biosynthesis of unsaturated fatty acids, bile secretion, and ABC transporters. The metabolites 2‐Hydroxy‐6‐Aminopurine, 17α‐Hydroxyprogesterone, and *S*‐(methyl) glutathione have been identified as potential metabolic biomarkers linked to the pathogenesis of AMI. The diagnostic model that integrates these three metabolites exhibited exceptional performance in both the discovery and validation cohorts, attaining an area under the curve (AUC) value greater than 0.9. In addition, based on the follow‐up data, we also found that the three metabolites were potential predictive biomarkers for poor prognosis of AMI. This study delineated the characteristic metabolic profile of AMI and assessed the value of metabolic molecules in the diagnosis and prognosis of AMI. This may provide insights for understanding the AMI occurrence and progression.

## Graphical abstract

This study detected 1498 metabolites using untargeted metabolomics in discovery and validation sets. Multivariate analysis revealed differences between healthy controls, AMI, and UA groups, identifying AMI‐specific metabolites. Enrichment analysis explored their biological significance. Machine learning techniques like random forest and neural networks identified high‐potential biomarkers, validated in an independent set for reliability.

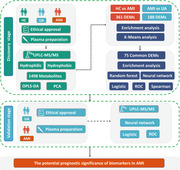

## 1 Introduction

Cardiovascular diseases (CVDs) continue to represent the predominant health threat and persist as the leading cause of mortality on a global scale [1]. Acute myocardial infarction (AMI) is the most common and fatal CVD caused by necrosis of the cardiac myocytes due to prolonged ischemia and hypoxia, characterized by high incidence, with more than 7 million newly diagnosed cases per year [2]. The World Health Organization (WHO) reported that AMIs account for approximately 31% of the total deaths globally. In China, the number of hospital admissions for AMI has increased more than fourfold, and the age of onset tends to be younger [3]. In addition, with the improvement of economic conditions and the intensification of aging, the prevalence of hypertension and hyperlipidemia is steadily rising in China [4]. This further leads to an increase in the incidence of AMI. Once AMI is established, it progresses rapidly and results in high mortality rates [5]. Research has demonstrated that early identification, expedited diagnostic procedures, and timely intervention can significantly reduce mortality rates and enhance the prognosis for patients with AMI. Therefore, it is crucial to identify novel biomarkers that can help diagnose AMI and improve AMI prognoses.

The clinical presentations of AMI patients, including chest tightness and/or persistent chest pain accompanied by dyspnea, are similar to that of unstable angina (UA) patients, and UA is often classified as a precursor of AMI. Currently, the identification of AMI primarily depends on the presence of irregularities in the electrocardiogram and increased concentrations of cardiac biomarkers [6], and the latter indicates irreversible damage to the cardiac myocytes, which requires urgent identification of novel diagnostic biomarkers of AMI. Metabolomics is regarded as an emerging discipline that belongs to the downstream system of biology and can reflect the metabolic processes of organisms [7]. In recent years, small molecule metabolites have shown excellent clinical application potential in the diagnosis of diseases [8]. Metabolic abnormalities are also the most prominent features of CVDs. Utilizing a high‐resolution metabolomics platform, Adnan Khan and colleagues discovered that tryptophan, carnitine, L‐homocysteine sulfinic acid, and cysteic acid serve as promising biomarkers for differentiating AMI patients from healthy controls (HC) [9]. Utilizing ultra‐performance liquid chromatography coupled with a quadruple time‐of‐flight mass spectrometry platform, Park et al. discerned notable disparities in lipid metabolism profiles between patients diagnosed with AMI and those suffering from UA [10]. It has been shown that small molecule metabolites may not only serve as biomarkers for disease diagnosis and prognosis but can also act as active drivers in biological processes and may participate in the regulation of the transcriptome and metabolome levels [8, 11]. Therefore, outlining the distinct metabolic profiles may aid in elucidating the underlying mechanisms of pathogenesis and in discovering novel therapeutic targets for AMI.

Nevertheless, the metabolic profiles associated with AMI and UA remain inadequately understood. In the present investigation, we conducted a comprehensive metabolomic analysis utilizing plasma samples obtained from 120 patients diagnosed with CVDs of various etiologies alongside 30 HC subjects. This analysis was executed employing an Ultra Performance Liquid Chromatography Tandem Mass Spectrometry (UPLC‐MS/MS) platform in conjunction with targeted detection methodologies. In addition, multiple machine learning algorithms were applied to identify the potential metabolome biomarkers associated with AMI prognosis, which were externally validated. There might be new insights into the pathogenesis and diagnosis of AMI based on this observation.

## 2 Results

### 2.1 Identification of Full Spectrum Metabolites

**FIGURE 1:**
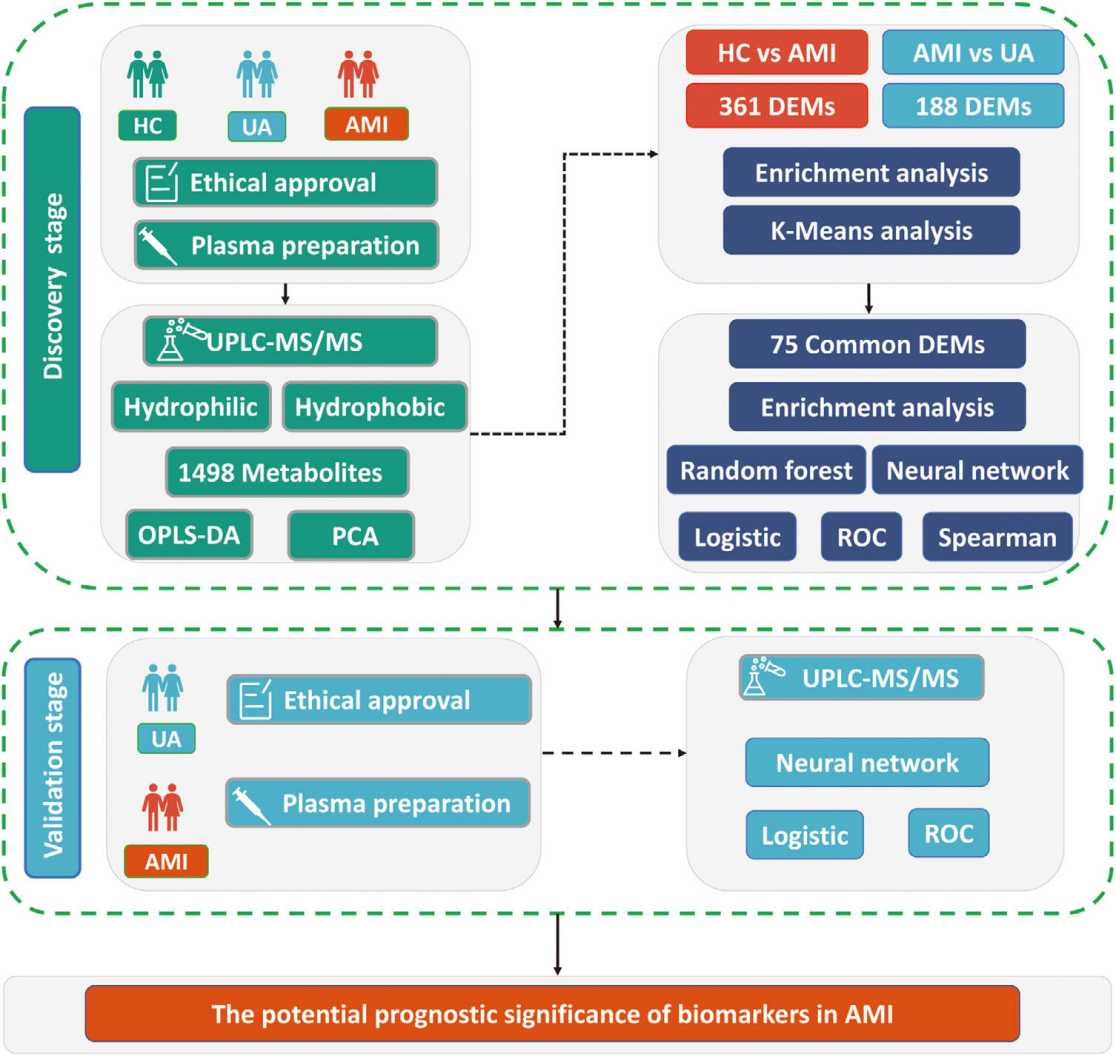
The workflow chart for this study.

The study included a total of 150 participants, who were divided into two distinct groups: the discovery cohort and the validation cohort (Table 1). A thorough analysis identified a total of 1498 metabolites, comprising both hydrophilic and hydrophobic varieties, through the application of the UPLC‐MS/MS platform in conjunction with advanced targeted lipid quantification methodologies. The detailed design process of this study is shown in the flow diagram (Figure 1).

**TABLE 1:** The clinical data and laboratory test results of all patients.

		Discovery set (*n* = 90)			Validation set (*n* = 60)	
Characteristic		HC (*n* = 30)	UA (*n* = 30)	AMI (*n* = 30)	UA (*n* = 30)	AMI (n = 30)
Age	≤ 60	14	13	14	13	15
	> 60	16	17	16	17	15
Sex	Male	21	21	23	21	23
	Female	9	9	7	9	7
CK‐MB			15.35 (13.43, 18.48)	54.45 (23.77, 194.47)	13.75 (11.03, 17.88)	37.5 (24.6, 160.8)
TC			4.88 ± 0.86	4.85 ± 1.05	4.57 ± 1.06	5.13 ± 1.25
TG			1.77 ± 1.31	1.34 ± 0.84	1.48 ± 0.66	1.57 ± 1.14
HDL‐C			1.23 ± 0.29	1.27 ± 0.73	1.15 ± 0.21	1.13 ± 0.23
LDL‐C			2.88 ± 0.79	2.94 ± 0.9	2.65 ± 0.81	3.1 ± 1.04

Abbreviations: AMI, acute myocardial infarction; CK‐MB, creatine kinase‐MB; HC, healthy control; HDL‐C, high‐density lipoprotein cholesterol; LDL‐C, low‐density lipoprotein cholesterol; TC, total cholesterol; TG, triglycerides; UA, unstable angina.

### 2.2 Identification of Metabolites With Differential Expression

**FIGURE 2:**
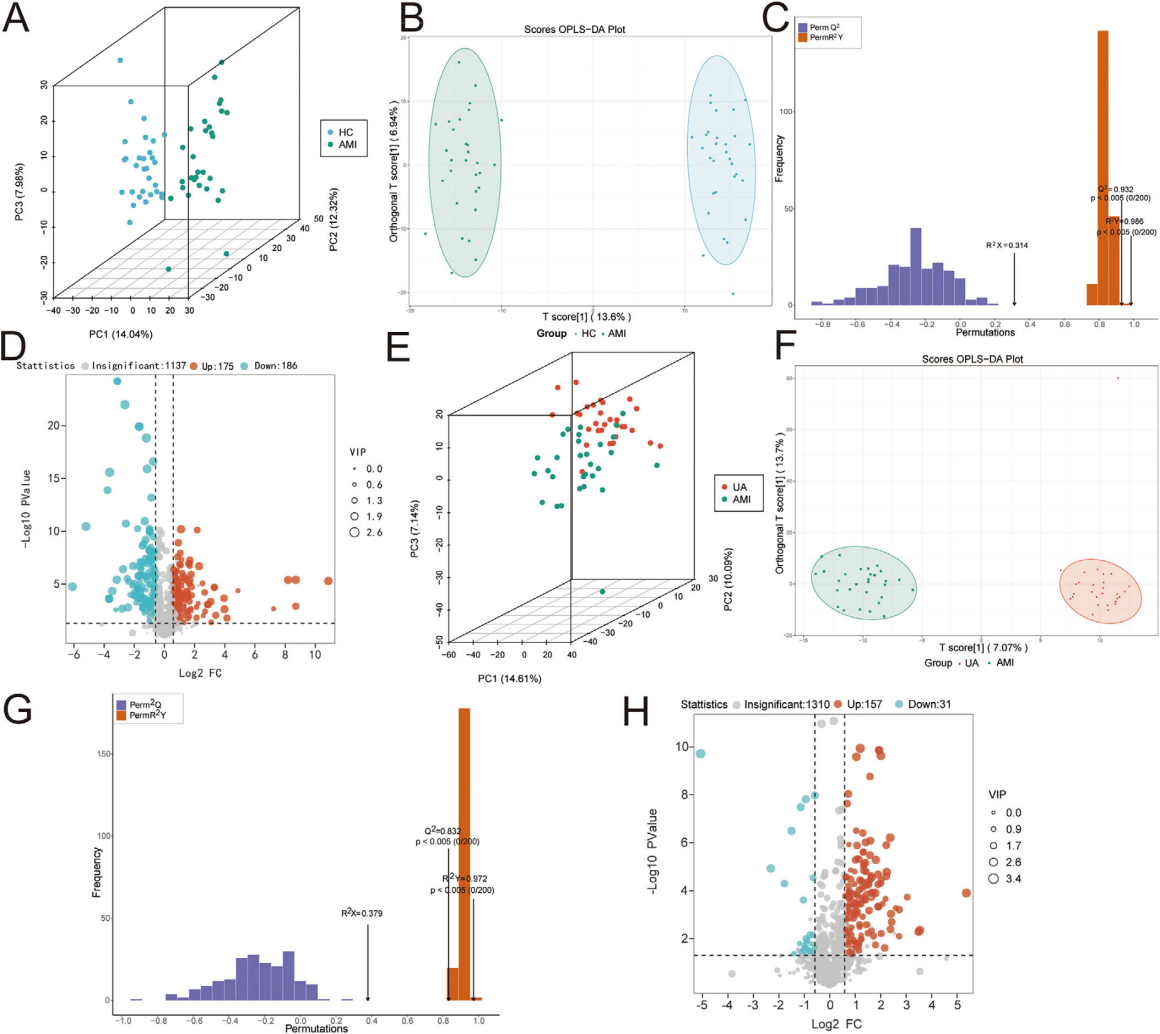
The serum metabolic profiles of the acute myocardial infarction (AMI) group, unstable angina (UA), and the healthy control (HC) group. (A) The PCA scores plot showed the separation trend between the AMI group and the HC group. (B) OPLS‐DA plot was used to characterize the differential metabolites between the AMI group and the HC group. (C) A permutation test was applied 200 times to assess the risk of over‐fitting for the OPLS‐DA model. (D) Volcano plot displaying the differences in metabolite concentrations between the AMI group and the HC group. (E) PCA scores plot showed the separation trend between the AMI group and the UA group. (F) OPLS‐DA plot was used to characterize the differential metabolites between the AMI group and the UA group. (G) A permutation test was applied 200 times to assess the risk of over‐fitting for the OPLS‐DA model. *R* ^2^ *X* and *R* ^2^ *Y*, respectively, represent the interpretation rate of the model to the X and Y matrices, and *Q* ^2^ represents the prediction ability of the model. (H) Volcano plot displaying the differences in metabolite concentrations between the AMI group and the UA group.

Principle component analysis (PCA) showed the separation between AMI, UA, and HC groups (Figure 2A), which reveals the considerable changes in the metabolome profile during AMI development. The score plot of the orthogonal partial least squares discriminant analysis (OPLS‐DA), as depicted in Figure 2B, demonstrates a significant distinction in metabolite profiles between the AMI group and the HC group. The permutations test evaluated the OPLS‐DA model's robustness by calculating *R* ^2^ and *Q* ^2^ values. With *Q* ^2^ = 0.932 and *R* ^2^ *Y* = 0.986, the model is reliable and stable, effectively identifying differential metabolites (Figure 2C). The identification of differential metabolites between the two groups was carried out utilizing the variable importance in projection (VIP) value, fold change, and *p* value, with the results subsequently illustrated in a volcano plot. In the AMI group, 175 metabolites were upregulated, and 186 were downregulated (Figure 2D). We also compared metabolite differences between the UA and AMI groups. PCA showed obvious spectral separation between the two groups and a high degree of consistency within groups (Figure 2E). Similarly, the OPLS‐DA plot showed that there were significantly different metabolic characteristics between the UA group and the AMI group (Figure 2F). The permutation analysis validated the robustness and reliability of the OPLS‐DA model, yielding *R* ^2^ *Y* and *Q* ^2^ values of 0.972 and 0.832, respectively (Figure 2G). A comprehensive evaluation revealed that a total of 188 metabolites exhibited differential expression across the groups, with 157 metabolites demonstrating an increase and 31 showing a decrease in the AMI cohort (Figure 2H). We analyzed and compared the differing metabolite profiles between the UA and HC groups. Employing PCA and OPLS‐DA, we identified a distinct separation in the metabolic profiles of these two cohorts (Figure S1A, B). The volcano plot analysis indicated that 86 metabolites were upregulated, whereas 209 metabolites were downregulated in the UA group relative to the HC group (Figure S1C). Moreover, the KEGG enrichment analysis revealed that these metabolites exhibiting significant differences were predominantly concentrated within various metabolic pathways, such as thermogenesis, nucleotide metabolism, and regulation of the actin cytoskeleton (Figure S1D). Subsequently, we conducted a comprehensive analysis by integrating the metabolic data from the three groups. PCA revealed a significant differentiation between the AMI and UA groups compared to the HC group, although some overlap was observed in the metabolic profiles of the AMI and UA groups relative to the HC group (Figure S2A). OPLS‐DA further demonstrated significant differentiation in the metabolic profiles among the three groups. Additionally, the credibility of the model was confirmed through a permutation test (Figure S2B,C). An ANOVA test was employed to determine the primary differential metabolites among the three groups, with a significance threshold set at *p* < 0.05. Furthermore, KEGG enrichment analysis revealed that these differential metabolites were predominantly associated with pathways such as thermogenesis, pyrimidine metabolism, nucleotide metabolism, and glyoxylate and dicarboxylate metabolism, among others (Figure S2D).

### 2.3 Metabolic Pathways Enrichment Analysis

**FIGURE 3:**
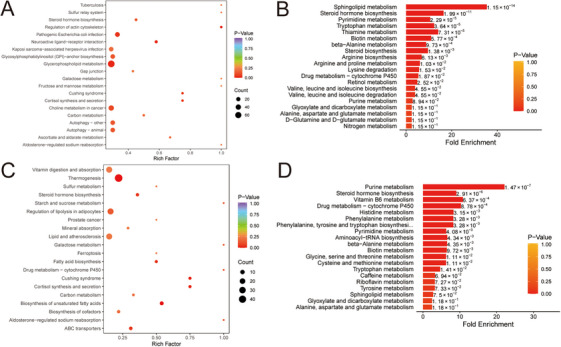
Enrichment analysis of differential metabolites. (A) Kyoto Encyclopedia of Genes and Genomes (KEGG) enrichment analysis of differential metabolites between acute myocardial infarction (AMI) and healthy control (HC). (B) Metabolic set enrichment analysis between AMI and HC. (C) KEGG enrichment analysis of differential metabolites between AMI and unstable angina (UA). (D) Metabolic set enrichment analysis between AMI and UA.

The KEGG pathway enrichment analysis performed on the differential metabolites differentiating the HC group from the AMI group identified significant enrichments in pathways related to pathogenic *Escherichia coli* infection, glycerophospholipid metabolism, and autophagy processes (Figure 3A). Metabolite set enrichment analysis (MSEA) showed that sphingolipid metabolism, steroid hormone biosynthesis, pyrimidine metabolism, tryptophan metabolism, thiamine metabolism, biotin metabolism, and beta‐alanine metabolism were significantly enriched between the HC group and the AMI group (Figure 3B). KEGG analysis revealed that the metabolites exhibiting significant differences between the UA and the AMI group were primarily enriched in thermogenesis, biosynthesis of unsaturated fatty acids, cortisol synthesis and secretion, Cushing syndrome, fatty acid biosynthesis, steroid hormone biosynthesis, and ABC transporters (Figure 3C). MESA revealed notable differences between the UA and AMI groups in purine metabolism, steroid hormone biosynthesis, vitamin B6 metabolism, histidine metabolism, and phenylalanine metabolism pathways (Figure 3D).

### 2.4 K‐Means Analysis

**FIGURE 4:**
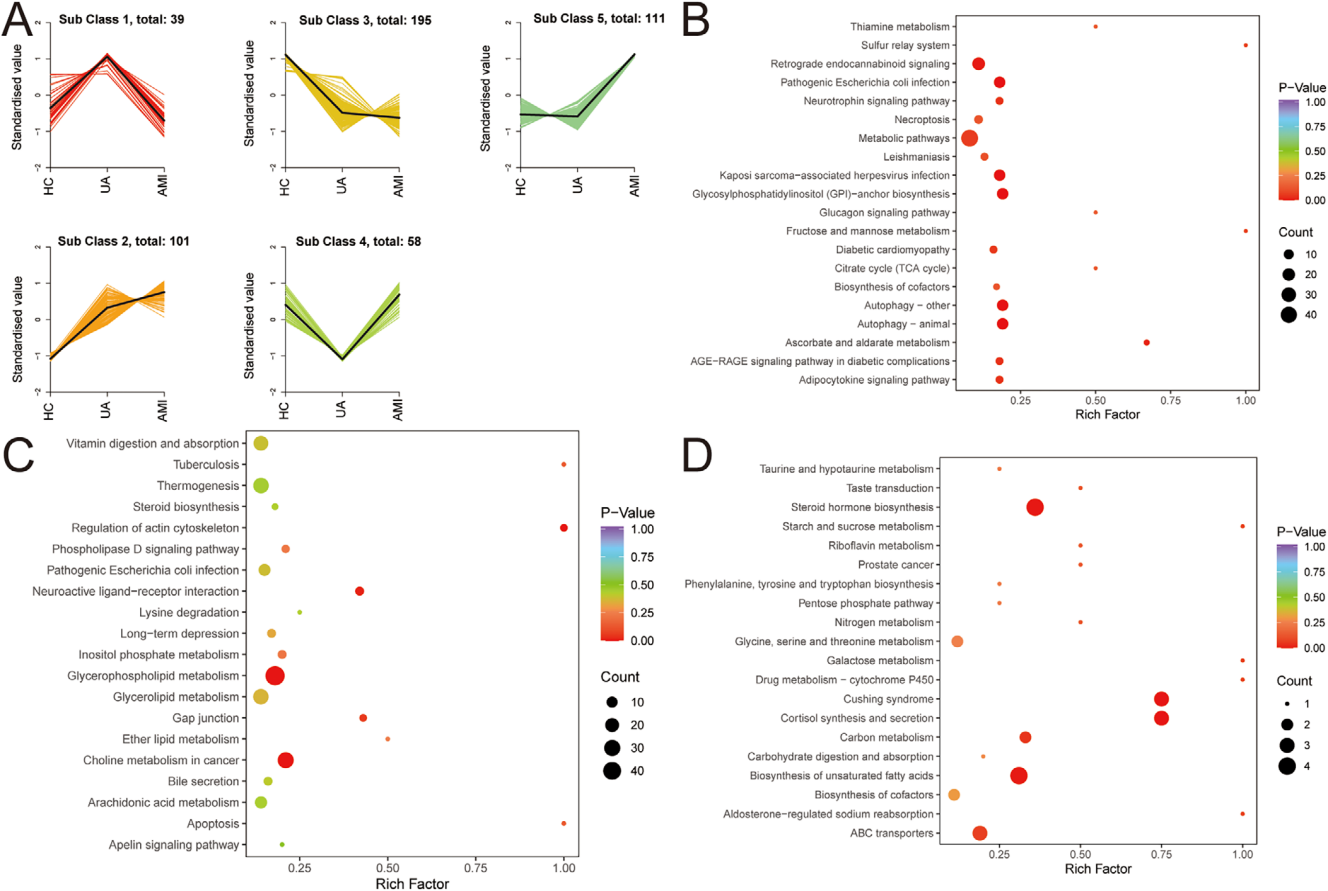
K‐means clustering and enrichment analysis. (A) The cluster analysis of differential metabolites was performed through K‐means clustering algorithms. (B) KEGG enrichment analysis of metabolites in Subclass 2. (C) KEGG enrichment analysis of metabolites in subclass 3. (D) KEGG enrichment analysis of metabolites in subclass 5. Acute myocardial infarction; AMI, unstable angina; UA, healthy control: HC.

We conducted K‐means clustering to examine metabolite trends across three groups, identifying five subclasses (Figure 4A). Subclasses 2, 3, and 5 showed consistent trends, either increasing or decreasing, from the HC group to the UA group and then to the AMI group. Then, we performed KEGG enrichment analysis on the metabolites in these three classes to understand their potential functions. The metabolites in subclass 2 were mainly enriched in retrograde endocannabinoid signaling, the neurotrophy signaling pathway, glycosylphosphatidylinositol‐anchor biosynthesis, diabetic cardiomyopathy, and autophagy pathways (Figure 4B). Subclass 3 metabolites were primarily enriched in actin cytoskeleton regulation, neuroactive ligand–receptor interaction, glycerophospholipid metabolism, and choline metabolism in cancer pathways (Figure 4C). Steroid hormone biosynthesis, Cushing syndrome, cortisol synthesis and secretion, biosynthesis of unsaturated fatty acids, ABC transporters, and other pathways were significantly enriched in subclass 5 (Figure 4D).

### 2.5 Screening of Potential Biomarkers for AMI

**FIGURE 5:**
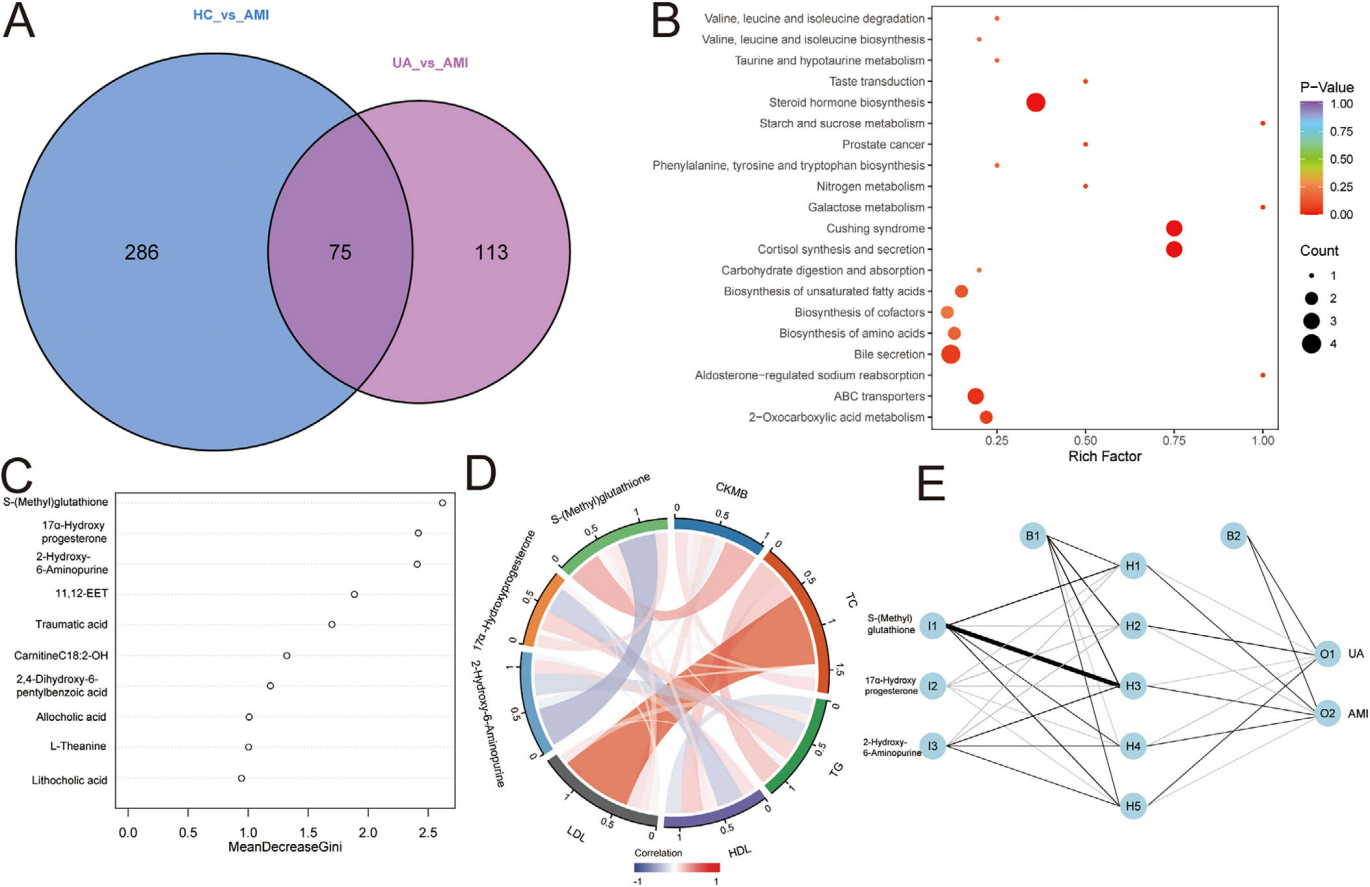
Screening and identification of serum biomarkers. (A) Venn diagram showing the common differential metabolites. (B) KEGG enrichment analysis of common differential metabolites. (C) Identification of the top 10 important metabolites in distinguishing between the acute myocardial infarction (AMI) group and the unstable angina (UA) group by random forest algorithm. (D) Pearson correlation analysis was used to evaluate the correlation between metabolites and traditional clinical indicators. (E) Visualization results of artificial neural network.

We used a Venn diagram analysis to identify metabolites unique to the AMI group. The results showed that 74 metabolites were identified as common differential metabolites of the HC group and the AMI group and the UA group and the AMI group, of which 64 metabolites were upregulated and 10 were downregulated (Figure 5A). KEGG enrichment pathway analysis showed that these common differential metabolites were mainly enriched in steroid hormone biosynthesis, Cushing syndrome, cortisol synthesis and secretion, bile secretion, and ABC transporters (Figure 5B). A random forest algorithm was applied to screen the key biomarkers for distinguishing between the UA group and the AMI group. The importance of predictors in the constructed regression trees was ranked, and the top 10 metabolites were *S*‐(Methyl) glutathione, 17α‐Hydroxyprogesterone, 2‐Hydroxy‐6‐Aminopurine, 11,12‐EET, Traumatic acid, Carnitine C18:2‐OH, 2,4‐Dihydroxy‐6‐pentylbenzoic acid, Allocholic acid, L‐Theanine, and Lithocholic acid (Figure 5C). Metabolites with relative importance greater than 2 were considered as potential biomarkers for AMI, including *S*‐(Methyl) glutathione, 17α‐Hydroxyprogesterone, and 2‐Hydroxy‐6‐Aminopurine. Serum creatine kinase‐MB (CK‐MB) enzyme is often used in clinical diagnosis of AMI. Total cholesterol (TC), triglyceride (TG), high‐density lipoprotein cholesterol (HDL‐C), and low‐density lipoprotein cholesterol (LDL‐C) are commonly used as risk factors for CVDs. In the present study, we found that the correlation between the three metabolites and commonly used clinical indicators was weak (Figure 5D). Subsequently, we defined minimum (0) and maximum values (1) based on the median expression level of the three metabolites in the discovery set, and the hidden layers’ number was 5 to build an artificial neural network (ANN) (Figure 5E).

**FIGURE 6:**
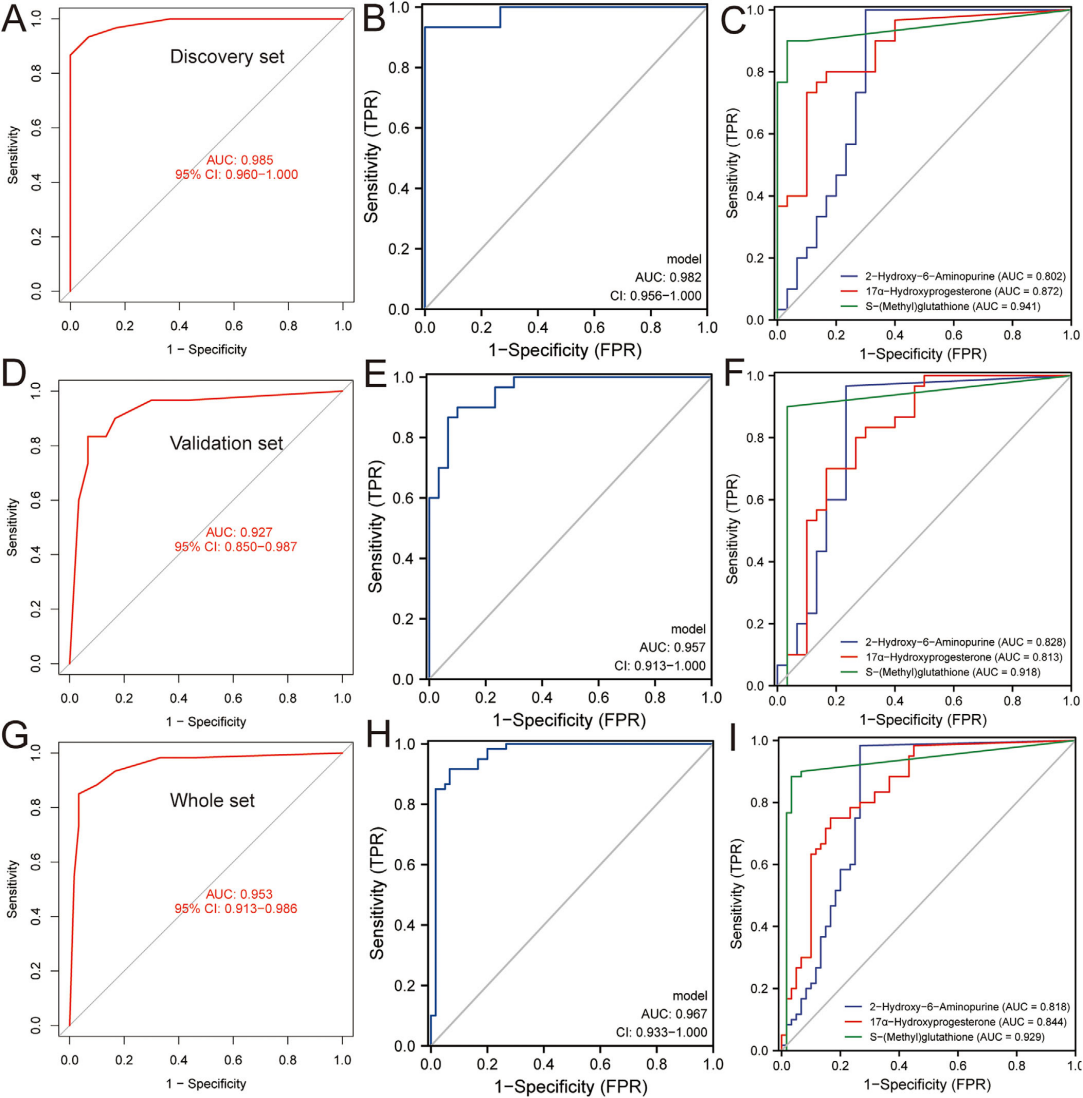
ROC analysis of diagnostic biomarkers. (A) ROC analysis of the artificial neural network model in the discovery set. (B) ROC analysis of the logistic regression model in the discovery set. (C) Diagnostic efficacy of the three metabolites in the discovery set. (D) ROC analysis of the artificial neural network model in the validation set. (E) ROC analysis of the logistic regression model in the validation set. (F) Diagnostic efficacy of the three metabolites in the validation set. (G) ROC analysis of the artificial neural network model in the whole set. (H) ROC analysis of the logistic regression model in the whole set. (I) Diagnostic efficacy of the three metabolites in the whole set.

Receiver operating characteristic (ROC) analysis indicated the model's strong diagnostic accuracy with an area under the curve (AUC) of 0.985 (Figure 6A). Additionally, a logistic regression model for the three metabolites in the discovery cohort achieved an AUC of 0.982 (Figure 6B). Subsequently, the prediction efficacy of these three metabolites was evaluated in the discovery set. ROC analysis indicated that 2‐Hydroxy‐6‐Aminopurine, 17α‐Hydroxyprogesterone, and *S*‐(Methyl) glutathione were promising biomarkers with AUC of 0.802, 0.872, and 0.941, respectively (Figure 6C). In the validation set, we also established the ANN model and logistic regression model of the three biomarkers, with an AUC of 0.927 and 0.957, respectively (Figure 6D,E). Similarly, these three metabolic biomarkers (2‐hydroxy‐6‐aminopurine, 17α‐Hydroxyprogesterone, and *S*‐(methyl) glutathione) also showed good diagnostic efficacy in the validation set, with AUC of 0.828, 0.813, and 0.918, respectively (Figure 6F). To evaluate the potential of three metabolites as AMI diagnostic biomarkers, we merged the discovery and validation sets. The metabolites effectively distinguished between the AMI and UA groups, with the ANN model achieving an AUC of 0.953 and the logistic regression model an AUC of 0.957 (Figure 6G,H). The 2‐Hydroxy‐6‐Aminopurine, 17α‐Hydroxyprogesterone, and *S*‐(Methyl) glutathione had strong robustness and stable prediction performance in the whole set, with AUC of 0.818, 0.844, and 0.929, respectively (Figure 6I).

### 2.6 Correlation Analysis Between Clinical Features and Metabolites

To clarify the diagnostic value of the three metabolites in AMI, we conducted univariate and multivariate logistic regression analyses. Univariate analysis identified CK‐MB and the three metabolites as AMI risk factors in the discovery set (Table 2). Multivariate analysis showed these metabolites as independent AMI risk factors, separate from CK‐MB (Table 2). The validation set confirmed these metabolites as independent AMI risk factors through both analyses (Table 3).

**TABLE 2:** Univariate and multivariate logistic regression in the discovery set.

	Univariate analysis		Multivariate analysis	
Characteristics	OR (95% CI)	*p* value	OR (95% CI)	*p* value
CK‐MB	1.132 (1.040–1.233)	0.004	NS	NS
TC	0.970 (0.567–1.660)	0.913	NS	NS
TG	0.677 (0.404–1.137)	0.14	NS	NS
HDL‐C	1.156 (0.448–2.979)	0.765	NS	NS
LDL‐C	1.081 (0.589–1.986)	0.801	NS	NS
2‐Hydroxy‐6‐Aminopurine	1.260 (1.106–1.435)	< 0.001	1.222 (1.055–1.416)	0.008
17α‐Hydroxyprogesterone	1.386 (1.172–1.639)	< 0.001	1.306 (1.081–1.578)	0.006
*S*‐(Methyl)glutathione	1.983 (1.448–2.715)	< 0.001	1.702 (1.224–2.367)	0.002

Abbreviations: CK‐MB, creatine kinase‐MB; HDL‐C, high‐density lipoprotein cholesterol; LDL‐C, low‐density lipoprotein cholesterol; NS, no significance; OR, odds ratio; TC, total cholesterol; TG, triglycerides.

**TABLE 3:** Univariate and multivariate logistic regression in the validation set.

	Univariate analysis		Multivariate analysis	
Characteristics	OR (95% CI)	*p* value	OR (95% CI)	*p* value
CK‐MB	1.145 (1.049–1.249)	0.002	NS	NS
TC	1.546 (0.959–2.490)	0.074	NS	NS
TG	1.107 (0.632–1.939)	0.723	NS	NS
HDL‐C	0.677 (0.064–7.146)	0.746	NS	NS
LDL‐C	1.724 (0.951–3.128)	0.073	NS	NS
2‐Hydroxy‐6‐Aminopurine	1.339 (1.159–1.546)	< 0.001	1.320 (1.112–1.567)	0.002
17α‐Hydroxyprogesterone	1.306 (1.107–1.540)	0.002	1.239 (1.040–1.475)	0.016
*S*‐(Methyl)glutathione	1.502 (1.235–1.827)	< 0.001	1.286 (1.060–1.560)	0.011

Abbreviations: CK‐MB, creatine kinase‐MB; HDL‐C, high‐density lipoprotein cholesterol; LDL‐C, low‐density lipoprotein cholesterol; NS, no significance; OR, odds ratio; TC, total cholesterol; TG, triglycerides.

**FIGURE 7:**
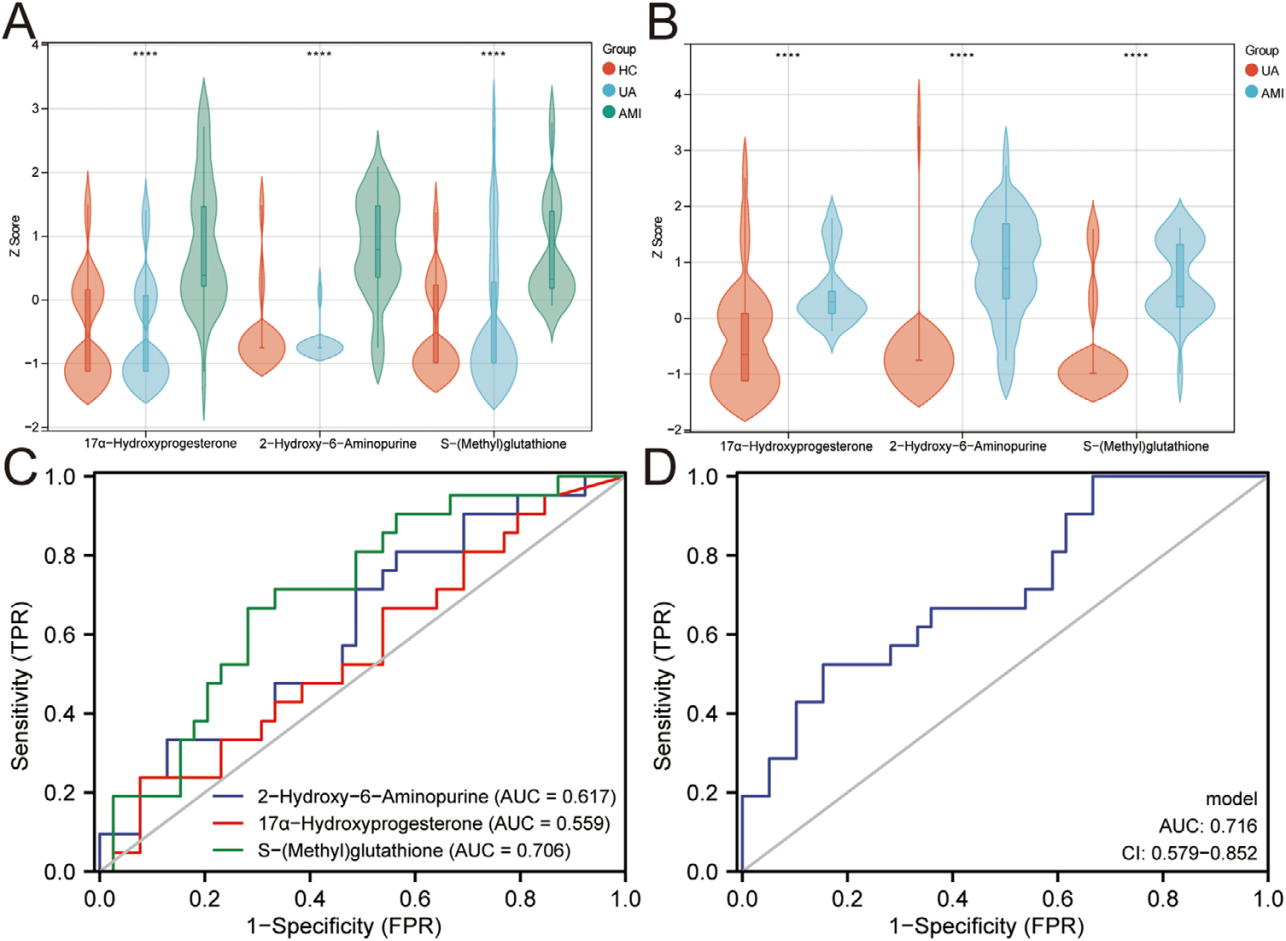
Expression and prognosis analysis of metabolites. (A) Expression of the three metabolites in the discovery set. (B) Expression of the three metabolites in the discovery set. The expression levels were log2 and *Z*‐score normalized. (C) Prognostic efficacy of the three metabolites in AMI. (D) ROC analysis of logistic regression model. ****p < 0.0001.

We analyzed the expression levels of three metabolites in the HC and AMI groups, finding significant upregulation in the AMI group in both the discovery and validation sets (Figure 7A,B). To assess their prognostic value, 60 AMI patients were followed for 18 months, with 21 (35%) hospitalized for CVDs. ROC analysis indicated these metabolites could predict second hospitalizations in AMI patients, with AUCs of 0.617, 0.559, and 0.706, respectively (Figure 7C). Univariate and multivariate logistic regression analyses indicate that *S*‐(Methyl) glutathione could be an independent prognostic factor, regardless of CK‐MB levels (Table S1). Furthermore, we constructed a logistic regression model for the three metabolites, and the AUC value reached 0.716 (Figure 7D).

## 3 Discussion

AMI has attracted widespread attention for its high incidence rate and mortality [2]. Over 9 million people worldwide die annually from AMI, the leading cause of death for both genders. The rise of reperfusion therapy has improved AMI patient outcomes [12]. However, the optimal treatment period of AMI is within 6 h after onset. Early diagnosis is the core strategy to improve the treatment effect and prognosis of AMI [13]. Metabolites have shown great potential as biomarkers in a variety of complex diseases [14, 15]. Metabolomics is an important tool for large‐scale detection of metabolites and also has great potential to elucidate disease initiation and progression [8, 11, 16]. Previously, independent lipid metabolomics or nontargeted metabolomics have been used to explore the plasma metabolic profile of AMI [9, 17, 18, 19, 20]. In this study, 1498 metabolites were identified based on the UPLC‐MS/MS platform and targeted full‐spectrum metabolomics. The combination of quantitative lipidomics and widely targeted metabolomics is more conducive to systematically depicting the characteristic metabolic spectrum of AMI. In the present study, PCA and OPLS‐DA analysis indicated that the metabolic profile of the AMI group was significantly different from that of the HC and UA groups.

In this study, 361 significantly different metabolites were identified between the HC group and the AMI group. These differential metabolites covered many species, including amino acid and its metabolomics, bile acid, fatty acyl, hormones, and hormone‐related compounds. The changes in these metabolic pathways may be the potential risk factors for AMI, and monitoring the dynamic changes of related metabolites may be beneficial to the early prevention of AMI. In addition, we also compared the metabolic profiles between the UA group and the AMI group, and a total of 188 differential metabolites were identified. KEGG analysis revealed that purine metabolism, thermogenesis, fatty acid biosynthesis and biosynthesis, of unsaturated fatty acid pathways were significantly enriched. Changes in these pathways may also be related to the alteration of the ischemia and hypoxia microenvironment in patients with AMI [21]. Ischemia could induce the utilization of stored energy, including energy supply by lipolysis and activation of purine metabolism [22, 23, 24]. Furthermore, we also found that the ferroptosis pathway was enriched. Ferroptosis occurs due to the accumulation of lipid peroxides and eventually leads to iron‐dependent programmed cell death. Studies have shown that ferroptosis plays an important role in acute cardiac ischemic injury [25, 26, 27]. In this study, arachidonic acid was significantly upregulated in the AMI group and was associated with the activation of the ferroptosis pathway. Our results suggested that ferroptosis was activated in AMI at the level of metabolic pathways. Furthermore, we also found that ABC transporters, bile secretion, steroid hormone biosynthesis, cortisol synthesis, and secretion were specifically enriched in the AMI group compared with the HC and UA groups. ABC transporters are widely distributed in the outer membrane of cells and participate in the transmembrane transport of a variety of endogenous substances and xenobiotics, including lipid molecules, intracellular hormones, and other small molecules, by mediating ATP hydrolysis [28, 29]. Meanwhile, studies have reported that ABC transporters play a critical role in maintaining the balance of bile acid metabolism and cholesterol metabolism [30, 31, 32, 33]. Previous studies also reported that aberrant expression of ABC transporter‐related protein is closely related to atherosclerosis, chronic inflammation, and other diseases [34]. As we know, the occurrence of AMI is inseparable from the formation of atherosclerosis and the continuous stimulation of chronic inflammation. These results suggested that maintaining the stability of the ABC transporters metabolic pathway is essential for preventing AMI.

The random forest algorithm identified 2‐hydro‐6‐aminopurine, 17α‐hydroxyprogesterone, and *S*‐(methyl) glutathione as possible biomarkers for AMI. *S*‐(Methyl) glutathione, a derivative of amino acid metabolism, is mainly produced by the combination of halomethanes with glutathione under the catalysis of glutathione‐*S*‐transferase T1 [35]. The accumulation of halomethanes in the human body has been reported to be associated with the impairment of human immunity and the occurrence of cancer [36, 37, 38]. Our data showed that *S*‐(Methyl) glutathione was significantly enriched in the AMI group, indicating that halomethanes may be enriched in patients with AMI. 17α‐Hydroxyprogesterone is mainly produced in the adrenal cortex, and its accumulation in the body is primarily due to 21‐hydroxylase (CYP21A2) deficiency. In this study, the 21‐hydroxylase deficiency (CYP21) pathway was also significantly enriched in the AMI group. Studies have reported that the abnormal accumulation of 17α‐Hydroxyprogesterone in the body could increase the risk of CVD [39, 40]. However, the relationship between 17α‐Hydroxyprogesterone level and incident risks in subjects with AMI has not been investigated. Our data revealed that 17α‐Hydroxyprogesterone may be a potential biomarker for the diagnosis of AMI. 2‐hydroxy‐6‐aminopurine, also known as guanine, is one of the four constituent bases of nucleic acids. Guanine in the human body is low, but it plays an indispensable role in maintaining normal physiological function and normal metabolism. Guanine is especially prone to oxidative damage in vivo, producing oxidative guanine damage products, which can cause DNA damage [41, 42]. However, there are few reports about the abnormal accumulation of guanine in the body and its role in the occurrence of AMI. Our results suggested that guanine may be a risk factor for AMI, and further investigation is needed to clarify its role. Pearson correlation analysis indicated no significant correlation between these three metabolites and clinical risk indicators. The three metabolites were found to be independent risk factors for AMI through logistic regression analyses, and ROC analysis confirmed their strong predictive ability in both the discovery and validation sets, with AUC values over 0.8. These results suggested that the three metabolites could complement traditional clinical indicators and may serve as independent risk factors. We subsequently built a diagnostic model employing ANN and logistic regression, which exhibited excellent predictive ability in both the discovery and validation cohorts, achieving an AUC greater than 0.9. Our findings indicate that the three metabolites, particularly *S*‐(Methyl) glutathione, could be useful for predicting AMI prognosis and the likelihood of a second admission. Nevertheless, in comparison to the widely accepted biomarkers BNP and NT‐proBNP, the predictive value of the three AMI biomarkers identified in this study for heart failure requires further validation through larger cohort studies.

This study is subject to several limitations that warrant consideration. While this study incorporates both a discovery set and a validation set, further validation of the identified biomarkers in a larger cohort remains essential. Hypoxia in cardiomyocytes plays a crucial role in the pathogenesis of AMI, necessitating a deeper investigation into the relationship between the metabolic markers we identified and hypoxia. Moreover, the expression of these biomarkers in high‐risk populations and their implications for tertiary prevention warrant more comprehensive examination. The roles and potential mechanisms of these biomarkers in the process of infarctogenesis necessitate further validation through in vivo or in vitro experimentation.

This study utilizes UPLC‐MS/MS in conjunction with targeted full‐spectrum metabolomics to characterize the metabolic profile associated with AMI. Our findings revealed significant changes in glycerophospholipid metabolism, thermogenesis, steroid hormone biosynthesis, bile secretion, and ABC transporters between AMI and UA/HC subjects. The identified metabolome biomarkers 2‐hydroxy‐6‐aminopurine, 17α‐hydroxyprogesterone, and *S*‐(methyl) glutathione might be of great importance for the diagnosis and prognosis of AMI, identifying new potential targets for novel treatment approaches to decrease the high mortality rate of AMI.

## 4 Materials and Methods

### 4.1 Characteristics of Patients

In this research, blood samples collected from AMI patients (*n* = 60), UA (*n* = 60), and healthy normal volunteers (HC, *n* = 30) were subjected to metabolome analysis and phenotyping (AMI = 30; UA = 30; HC = 30) to identify the potential biomarkers associated with AMI pathogenesis. The findings were externally validated using an independent cohort (AMI = 30; UA = 30). Subjects with inheritable genetic disorders, infectious diseases, cancer, severe mental illness, and severe organ diseases were excluded. In this study, blood was collected from all patients within 6 h of onset, and none of the patients had taken any operative treatment. All participants were recruited from Yuebei People's Hospital. The ethical committee of Yue Bei People's Hospital, which is affiliated with Shantou University Medical College located in Shaoguan, China, granted ethical approval for the study design and protocol (KY‐2021‐050). Patients and volunteers were informed, and written consent was obtained. The framework and methodology employed in this investigation adhered to the principles established by the Declaration of Helsinki. Inclusion Criteria: Individuals identified with AMI in accordance with recognized clinical protocols. Exclusion Criteria: Individuals presenting with significant hypertension, respiratory issues, cardiac arrhythmias, hepatic and renal impairments, neoplastic conditions, and psychiatric disorders, as well as pregnant and breastfeeding women, in addition to those diagnosed with infectious and congenital diseases, were excluded from the study. Furthermore, we conducted a follow‐up study involving 60 patients with AMI over a duration of 18 months. The follow‐up endpoint was defined as hospitalization due to CVD during this period or follow‐up to 18 months.

### 4.2 Sample Processing and Metabolite Extraction

All samples were transported to the laboratory in coolers promptly following their collection, and plasma separation was carried out under sterile conditions. The plasma was sub‐packaged to the Eppendorf tube (200 µL per tube) and then stored in a −80°C refrigerator (TDE60086FV‐ULTS, Thermo) until further use. Samples were removed from the freezer and then transferred immediately into the icebox to facilitate gradual thawing, and all subsequent operations were conducted on ice. Plasma samples were completely thawed, followed by a vortex for 10 s to ensure adequate mixing. 50 µL of supernatant was absorbed from the mixed sample and transferred to a sterile and clean centrifuge tube. Subsequently, hydrophilic and hydrophobic metabolites were extracted according to the corresponding requirements. The extraction method for hydrophilic metabolites was as follows: (1) to each sample, 300 µL of extraction solution containing 20% acetonitrile (34851, Merck) methanol internal standard was directly transferred to 50 µL of plasma and then vortexed for 3 min to mix well. (2) Place the mixed samples into a centrifuge set at 4°C, and centrifuge at 12,000 r/min for 10 min. (3) Subsequent to centrifugation, the sample tube was placed in an icebox, and 200 µL of the supernatant was carefully transferred to a new centrifuge tube. This was followed by a resting period at −20°C for 30 min. (4) The samples were again centrifuged for 3 min at 4°C and 120,000 r/min. (5) After centrifugation, 180 µL of the supernatant was collected and subsequently placed into the inner liner of the injection vial for subsequent analysis. The extraction method for hydrophobic metabolites was as follows: (1) the 1 mL lipid internal standards (methyl‐*tert*‐butyl ether: methanol (34885, Merck) = 3:1, v/v) were added to the 50 µL sample, and vortexing was carried out for 15 min to fully mix the solution. (2) Next, 200 µL of ultrapure water was added to the mixed sample and vortexed thoroughly; the mixture was put into a centrifuge for 10 min at 4°C and 120,000 r/min after being mixed for 1 min. (3) About 200 µL of supernatants were recovered into a new sterile centrifugation tube, and the sample was then freeze‐dried with a concentrator (7310038, LABCONCO, Thermo). (4) A volume of 200 µL of mobile phase B, which consists of acetonitrile and isopropanol in a ratio of 10:90 (v/v) and is supplemented with 0.1% formic acid (F112034, Aladdin) and 10 mmol/L ammonium formate (A100186, Aladdin), was introduced into the lyophilization. This mixture was then thoroughly vortexed for a duration of 3 min and subsequently subjected to centrifugation at 4°C at a speed of 120,000 revolutions per minute. (5) After centrifugation, the supernatant was pipetted into autosampler vials for LC‐MS/MS analysis.

### 4.3 Chromatography and Mass Spectrometry Conditions

In the present investigation, data acquisition employed UPLC‐MS/MS. The conditions used for the analysis of hydrophilic metabolites and hydrophobic metabolites were different. The liquid phase conditions of hydrophilic metabolites were as follows:

*T3 liquid phase was constructed as follows*: (1) The chromatographic column employed in this study was the Waters ACQUITY UPLC HSS T3 C18, characterized by a particle size of 1.8 µm and dimensions of 2.1 mm in diameter by 100 mm in length; (2) Mobile phase A consisted of ultrapure water supplemented with 0.1% formic acid, while mobile phase B was composed of acetonitrile that also contained 0.1% formic acid; (3) The gradient elution procedure was conducted as follows: At the outset (0 min), a solvent mixture comprising water and acetonitrile in a 95:5 (v/v) ratio was employed. This composition was progressively altered to a 10:90 (v/v) ratio by 11.0 min, which was sustained until 12.0 min. Subsequently, at 12.1 min, the elution conditions were reverted to the initial 95:5 (v/v) ratio, which was maintained until 14.0 min; (4) The parameters for liquid chromatography were set as follows: a flow rate of 0.4 mL/min, a column temperature of 40°C, and a sample injection volume of 2 µL.

*The conditions for the liquid phase involving amides were described as follows*: (1) The chromatographic column utilized in this study was the Waters ACQUITY UPLC BEH Amide, featuring a particle size of 1.7 µm and dimensions of 2.1 mm in diameter by 100 mm in length; (2) Mobile phase A consisted of ultrapure water supplemented with 20 mM ammonium formate and 0.4% ammonium hydroxide, while mobile phase B was composed exclusively of pure acetonitrile; (3) A gradient elution was conducted starting with a 10:90 water‐acetonitrile mix at 0 min, shifting to 40:60 at 9 min, then to 60:40 at 10 min. This 60:40 mix was held until 11 min before returning to 10:90 at 11.1 min, lasting until the end at 15 min.

*The conditions for the liquid phase of hydrophobic metabolites were established as follows*: (1) The chromatographic column utilized in this study was a Thermo Accucore C30 with dimensions of 2.1 mm in inner diameter and a length of 100 mm, featuring a particle size of 2.6 µm. (2) The mobile phase A utilized in this study consisted of a mixture of acetonitrile and water in a 60:40 volume ratio, supplemented with 0.1% formic acid and 10 mmol/L ammonium formate. Conversely, mobile phase B was formulated using acetonitrile and isopropanol in a 10:90 volume ratio, also containing 0.1% formic acid and 10 mmol/L ammonium formate. (3) A gradient elution was conducted as follows: starting with a ratio of A/B (80:20, v/v) at 0 min, it changed to (70:30, v/v) at 2 min, (40:60, v/v) at 4 min, (15:85, v/v) at 9 min, and (10:90, v/v) at 14 min. At 15.5 min, it was adjusted to (5:95, v/v) and held until 17.3 min. The elution then reverted to (80:20, v/v) at 17.5 min, maintaining this until 20 min. (4) The liquid flow rate was 0.35 mL/min, the column was preheated to 45°C, and the injection volume was 2 µL.

*The mass spectrometric conditions of hydrophilic metabolites were as follows*: (1) the electrospray ionization temperature was set to 500°C, and the mass spectrometry voltage was configured to 5500 V in the positive ionization mode and −4500 volts in the negative ionization mode. The pressure of the ion source Gas I was maintained at 55 psi, while Gas II was set to 60 psi. Furthermore, the curtain gas (CUR) pressure was adjusted to 25 psi, and the collision‐induced dissociation parameter was designated as high. (2) Within the triple quadrupole system, each ion underwent scanning and detection through the application of optimized clustering potentials and collision energies.

*The mass spectrometric conditions of hydrophobic metabolites were as follows*: (1) The electrospray ionization process was conducted at a temperature of 500°C, utilizing a mass spectrum voltage of 5500 V in the positive mode and −4500 V in the negative mode. The pressure of the ion source Gas I was maintained at 55 psi, while Gas II was set at 60 psi. Additionally, the CUR pressure was established at 25 psi, and the collision‐activated dissociation was adjusted to a medium setting. (2) In the triple quadrupole system, the detection and scanning of each ion were conducted utilizing the optimized parameters for clustering potential and collision energy.

### 4.4 Data Processing and Analysis

The data produced were examined utilizing Analyst version 1.6.3 software. Metabolites were detected and compared based on the targeted standard database MWDB (Metware database), the duration for which the identified compounds are retained, and the details regarding the paired ions with double charge, and qualitative analysis was conducted on the secondary spectrum data. Triple quadrupole mass spectrometry in multi‐reaction monitoring mode was utilized to generate peak maps of detected substances. The chromatographic peak areas were scored and adjusted using MultiQuant software, followed by quantitative analysis through peak area integration.

### 4.5 Bioinformatics and Statistical Analysis

All statistical analyses of the data were performed utilizing R software (version 4.1.1), with the ggplot package employed for the purpose of data visualization. The Ropls package facilitated the execution of OPLS‐DA, subsequently allowing for the computation of the VIP of the metabolites. To identify differential metabolites, a combination of VIP, *p* value, and fold change derived from the univariate analysis was utilized. The established cut‐off criteria were set as follows: fold change greater than 1.5 or less than 0.66, *p* value below 0.05, and a VIP of at least 1. Furthermore, the MetaboAnalyst database (https://www.metaboanalyst.ca/) was employed to conduct MSEA. MSEA is a group‐based method that does not need preselected metabolites based on the specified threshold, which can avoid missing some metabolites with insignificant differential expression but important biological meaning. K‐means clustering was employed to examine the variations in metabolite levels across different groups. Random forest is a decision tree‐based method that was used to evaluate the importance of variables based on the randomForest package. The Spearman test was used to perform correlation analysis. ANN is a high‐dimensional, nonlinear, complex dynamic system. In this study, we first defined minimum and maximum values based on the median expression value, then constructed an ANN model using the “neuralnet” package, and the number of hidden layers was set to 5. Subsequently, the accuracy of the ANN model was evaluated using logistic regression analysis. The pROC software package was employed to construct ROC curves, followed by the calculation of the AUC values. To assess differences within the group, the Wilcoxon signed‐rank test was utilized, and statistical significance was determined with a *p* value threshold of below 0.05.

## Author Contributions

J.L.: writing of the article, serum collection, and data analysis. Y.Y.: writing – review and editing. Q.L.: serum collection and data analysis. S.‐Q.Z.: sample collection. J.‐C.L.: study design, writing – review and editing. All authors have read and approved the final manuscript.

## Ethics Statement

The study design and protocol were ethically approved by the ethics committee of the Yue Bei People's Hospital affiliated with Shantou University Medical College (KY‐2021‐050, China). Patients and volunteers were informed, and written consents were obtained.

## Conflicts of Interest

The authors declare no conflicts of interest.

## Supporting information



Supporting Information

## Data Availability

Data are available from the corresponding author on request.
