# Supplementary material for: Comprehensive Plasma Metabolome for Identification of Novel Biomarkers of Acute Myocardial Infarction
Source: MedComm (2020). 2025 Jul 27;6(8):e70303. doi: 10.1002/mco2.70303 (PMC12301166; doi:10.1002/mco2.70303)
Supplement: Supplementary file 1 — Supporting Information [file MCO2-6-e70303-s001.docx]

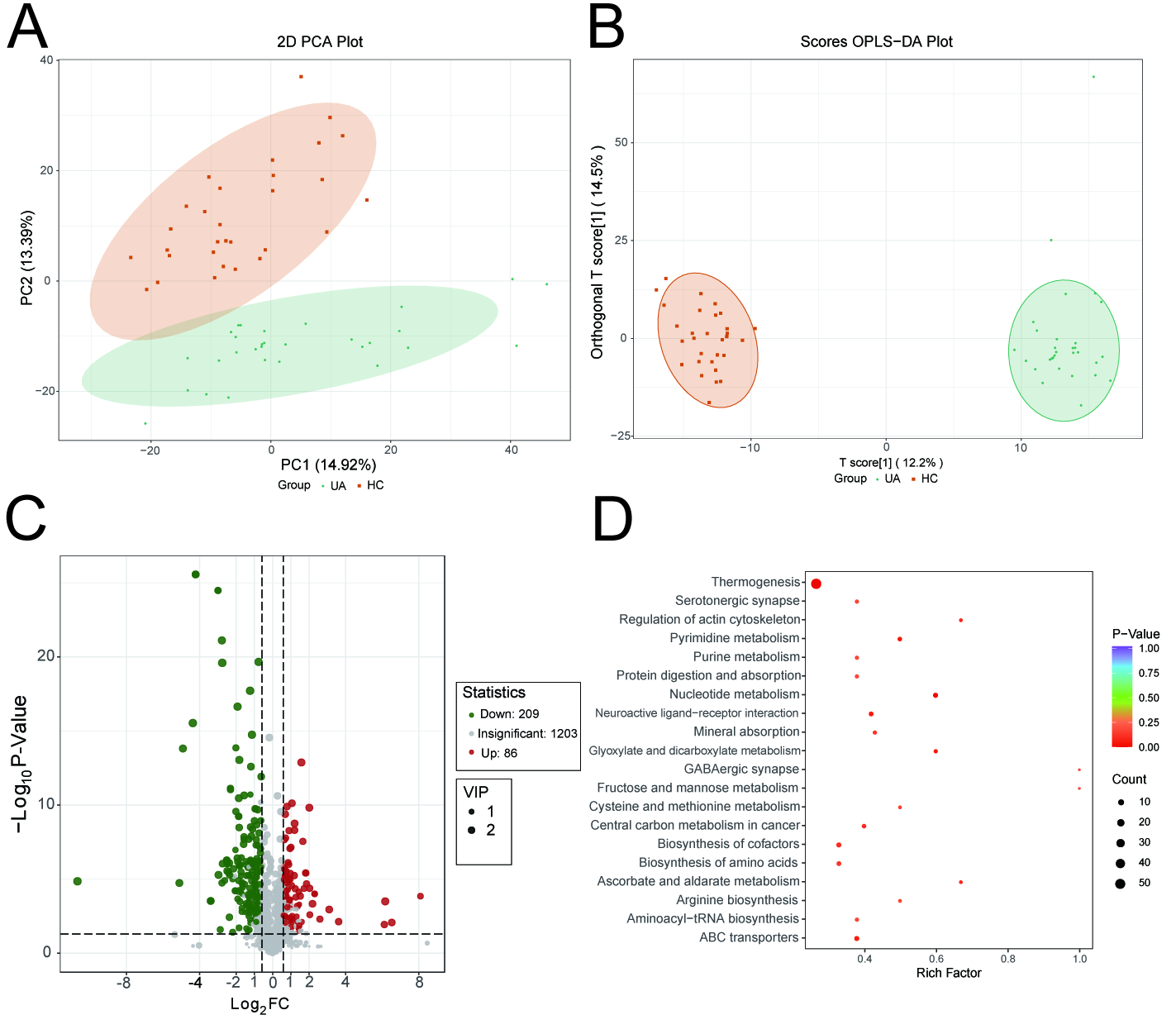


Figure S1. Metabolite analysis of differences between unstable angina (UA) patients and healthy controls (HC). A. PCA scores plot showed the separation trend between the UA group and the HC group. B. OPLS-DA plot was used to characterize the differential metabolites between the UA group and the HC group. C. Volcano plot displaying the differences in metabolite concentrations between the UA group and the HC group. D. KEGG enrichment analysis of differential metabolites between the UA group and the HC group.


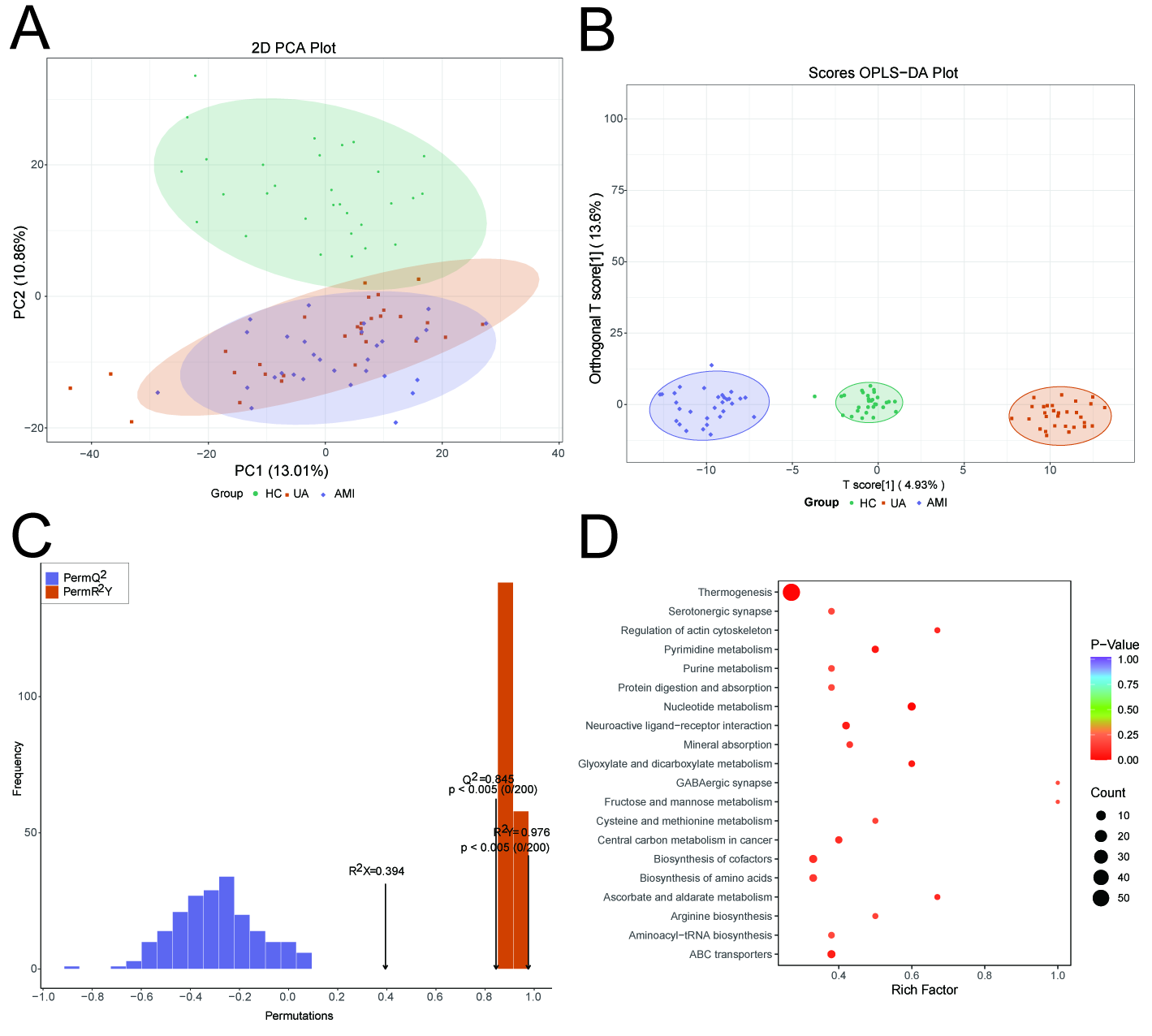


Figure S2. Integrated metabolite analysis of acute myocardial infarction (AMI), unstable angina (UA) patients, and healthy controls (HC). A. The Principal Component Analysis (PCA) scores plot demonstrated a separation trend among the AMI, UA, and HC groups. B. An Orthogonal Partial Least Squares Discriminant Analysis (OPLS-DA) plot was employed to characterize the differential metabolites among the AMI, UA, and HC groups. C. A permutation test was performed 200 times to evaluate the risk of overfitting in the OPLS-DA model. D. Kyoto Encyclopedia of Genes and Genomes (KEGG) enrichment analysis was conducted on the differential metabolites among the AMI, UA, and HC groups.

Table S1. Univariate and multivariate logistic regression analysis to evaluate the independent prognostic value.

| Characteristics | Total(N) | Univariate analysis | | Multivariate analysis | |
| --- | --- | --- | --- | --- | --- |
|  |  | Odds Ratio (95% CI) | P value | Odds Ratio (95% CI) | P value |
| CK-MB | 60 | 0.999 (0.996 – 1.003) | 0.630 |  |  |
| 2-Hydroxy-6-Aminopurine | 60 | 1.116 (0.972 – 1.281) | 0.119 |  |  |
| 17α-Hydroxyprogesterone | 60 | 1.044 (0.924 – 1.180) | 0.487 |  |  |
| S-(Methyl)glutathione | 60 | 1.174 (1.028 – 1.340) | 0.018 | 1.174 (1.028 – 1.340) | 0.018 |
